# Chemical Composition and Bioactivity of Extracts Obtained from *Prunus spinosa* Seeds by Supercritical CO_2_ Extraction

**DOI:** 10.3390/molecules30081757

**Published:** 2025-04-14

**Authors:** Alessandra Piras, Silvia Porcedda, Antonella Smeriglio, Domenico Trombetta, Franca Piras, Valeria Sogos, Antonella Rosa

**Affiliations:** 1 Department of Chemical and Geological Sciences, University of Cagliari, Cittadella Universitaria, SP 8, Monserrato-Sestu km 0.700, 09042 Monserrato, Italy; porcedda@unica.it; 2 Department of Chemical, Biological, Pharmaceutical and Environmental Sciences, University of Messina, Viale Ferdinando Stagno d’Alcontres 31, 98166 Messina, Italy; antonella.smeriglio@unime.it (A.S.); domenico.trombetta@unime.it (D.T.); 3 Department of Biomedical Sciences, University of Cagliari, Cittadella Universitaria, SS 554, km 4.5, 09042 Monserrato, Italy; fpiras@unica.it (F.P.); sogos@unica.it (V.S.)

**Keywords:** *Prunus spinosa*, supercritical extraction, phenolic compounds, fixed oil, fatty acids, cytotoxicity

## Abstract

This study investigates the potential reuse of *Prunus spinosa* (blackthorn) seeds, a food industry by-product. Traditionally discarded, these seeds are now being explored for their bioactive compounds. In this work, seeds were used as raw material for supercritical CO_2_ extraction. Two distinct extracts were obtained at low and high pressure (SFE90 and SFE200) and both extracts presented an aqueous phase (WE90 and WE200). SFE90 analysis by GC/MS allowed us to identify benzaldehyde and fatty acids (mainly oleic and linoleic acids). The fatty acid profile of SFE200, determined by HPLC-DAD/ELSD, showed that oleic and linoleic acids were predominant in supercritical oil. The phytochemical composition of the water extracts, analyzed via LC-DAD-ESI-MS, revealed that higher pressure enhanced the recovery of specific flavonols and anthocyanins, while lower pressure preserved various polyphenolic subclasses. WE90 was rich in 3-feruloylquinic acid and cyanidin-3-*O*-rutinoside, whereas WE200 was rich in caffeic acid hexoside 2 and dihydro-*o*-coumaric acid glucoside. Benzaldehyde was individuated in WE90 and WE200 by HPLC-DAD analysis. Cytotoxicity assays demonstrated that WE90, WE200 and SFE200 had anticancer effects on SH-SY5Y neuroblastoma cells, while all extracts did not remarkably affect the viability and morphology of human skin keratinocytes (HaCaT cells). These results suggest that *P. spinosa* seed extracts have potential nutraceutical and pharmaceutical applications.

## 1. Introduction

The Prunus genus, part of the Rosaceae family, includes around 200 species such as almonds, plums, apricots, peaches and cherries. These fruits are widely cultivated worldwide, with Europe being a major producer. Known for their popularity in temperate regions, Prunus species are valued not only for their flavor, color and sweetness but also for their nutritional benefits and bioactive compounds that support human health [1].

*Prunus spinosa* L., or blackthorn, is a deciduous shrub or small tree, commonly found in uncultivated areas, including the Mediterranean. Its small, bluish-black fruits have an astringent taste and are used in jams, jellies, juices, tea and alcoholic beverages, as well as for a natural food colorant [2]. Traditionally, it has been valued for its anti-inflammatory, diuretic, antispasmodic, laxative, antimicrobial and antioxidant properties [1,2,3,4]. It is used to treat inflammation-related disorders, metabolic diseases like diabetes and obesity, and circulatory issues [2]. Recent studies highlight its potential in wound healing, cancer cell cytotoxicity and selective antibacterial activity [2,4].

The processing of *P. spinosa* fruits for culinary and industrial applications—such as the production of sloe gin, jams and syrups—generates a significant quantity of by-products, particularly seeds [5]. Traditionally discarded, these seeds are increasingly being studied for their potential reuse in various industries, aligning with the principles of sustainability and waste reduction [6].

This research explores the composition, potential applications and challenges associated with using *P. spinosa* seeds as a by-product. Sloe seeds are small and hard, consisting of fatty acids (particularly oleic and linoleic acids, which is valuable in cosmetics and biofuels) [1,7,8], tocopherols [1,7,8], carotenoids [1], sterols [8], phenolic compounds (provide antioxidant properties, making the seeds useful in health supplements) [1,5,8], proteins (potential for nutritional applications) and cyanogenic glycosides (amygdalin and prunasin, present in small amounts, requiring careful processing to ensure safety) [1,5].

Supercritical carbon dioxide extraction (SFE-CO_2_) is a cutting-edge technique widely used for isolating bioactive compounds from natural sources [9,10,11]. SFE-CO_2_ extraction utilizes carbon dioxide above its critical temperature (31.1 °C) and pressure (73.8 bar), where it exhibits both gas-like diffusion and liquid-like solvating properties [12]. This makes CO_2_ an excellent solvent for extracting non-polar compounds, such as oils and lipids, while avoiding the use of toxic organic solvents. Due to its efficiency and environmentally friendly nature, this method is gaining attention for extracting oils from seeds [13], including *P. spinosa* seeds.

The effectiveness of supercritical CO_2_ extraction depends on several parameters [12,13,14]. Pressure typically ranges between 90 and 400 bar for seed oil extraction. Higher pressures increase oil yield but may require more energy. Temperature is generally maintained between 40 and 60 °C to protect thermolabile components. The CO_2_ flow rate ensures efficient extraction while balancing operational costs. Seeds are finely ground to reduce the particle size, increasing the surface area and extraction efficiency [12,13,14].

In this work, seeds of *P. spinosa* (blackthorn) were used as a raw material for supercritical CO_2_ extraction. The aim of this study was to obtain *P. spinosa* seed extracts under different pressure conditions, characterize the lipid fraction in terms of fatty acid (FA) composition, analyze the aqueous phase of the extracts for phenolic compounds and assess the bioactivity (cytotoxic effect) of the obtained extracts via an in vitro cell model. The advantages and process parameters of this technique for blackthorn seed extraction and potential applications of the obtained extracts were also explored.

The FA profile of extracted oil was characterized using a high-performance liquid chromatography system coupled with a photodiode array detector and evaporative light-scattering detector (HPLC-DAD/ELSD). The phytochemical profile of the aqueous phase (water extracts) was obtained by liquid chromatography–electrospray ionization–MS analysis (LC-DAD-ESI-MS analysis). The effect of water extracts and SFE-CO_2_ oil extract on cell viability and morphology (24 h of incubation) was tested in human skin HaCaT keratinocytes (a skin cell model) and SH-SY5Y human neuroblastoma cell line (cancer cells), cell lines amply used to assess the toxicity of natural extracts/compounds [15,16].

Literature data on the application of SFE-CO_2_ for *P. spinosa* extraction are scarce. SFE-CO_2_ has been widely used in oil extraction from several Prunus seeds, i.e., kernels of sweet cherry, sour cherry and apricot [5]. However, only one study has been found reporting the composition of the lipid fraction obtained by SFE-CO_2_ from *P. spinosa* seeds [17]. No information is available on the chemical composition or biological activity of supercritical extracts of *P. spinosa* seeds. In this context, we believe our findings play a significant role.

## 2. Results

### 2.1. Preparation of Extracts from P. spinosa Seeds and Extraction Yields

**Scheme 1:**
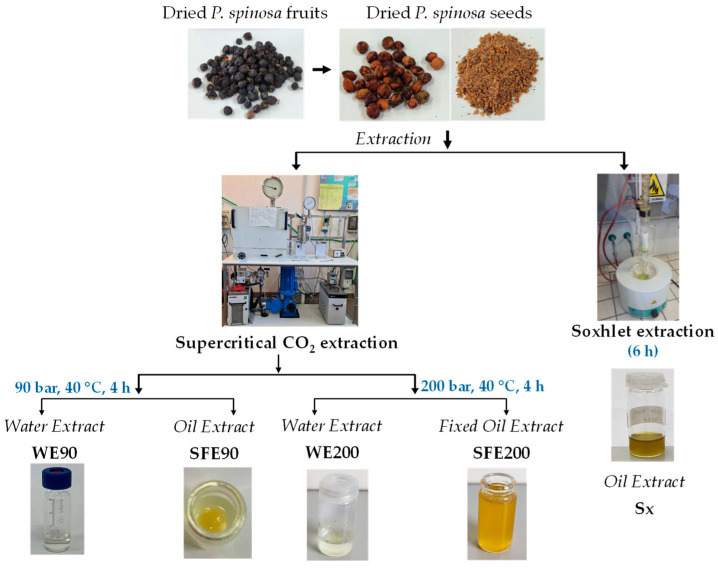
Scheme of preparation of extracts by supercritical CO_2_ extraction (WE90, SFE90, WE200 and SFE200) and Soxhlet extraction with *n*-hexane (Sx) obtained from dried seeds of *P. spinosa*.

Supercritical CO_2_ extraction was performed at two different pressures: 90 bar (low pressure) and 200 bar (high pressure) (Scheme 1). The temperature was maintained at 40 °C throughout the extraction process. CO_2_ was used as the supercritical fluid without additional solvents. Two distinct extracts were obtained at low and high pressure (SFE90 and SFE200) and both extracts presented an aqueous phase (WE90 and WE200), which was separated for further analysis.

The yield of the high-pressure extraction was higher than that of the low-pressure extraction (1.9% versus 1.2%) likely due to increased solvating power of CO_2_ at higher pressures. SFE200 was compared with n-hexane extraction using a Soxhlet apparatus (Sx oil extract, Scheme 1). As expected, the yield obtained from the latter was significantly higher, reaching 10.7%.

### 2.2. Chemical Composition of SFE90 as Determined by GC/MS

**Figure 1:**
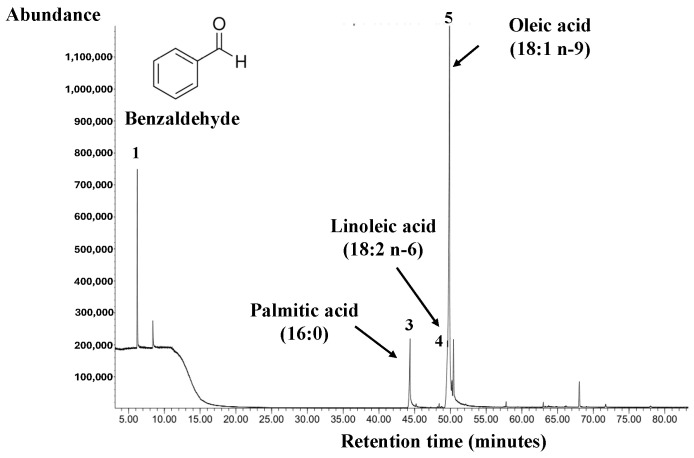
GC-MS chromatogram, obtained on an HP-5ms capillary column, of *P. spinosa* seed extract obtained by supercritical CO_2_ extraction at low pressure (SFE90).

Supercritical extract obtained at low pressure (SFE90) was analyzed using GC/MS (Agilent Technologies, Santa Clara, CA, USA) to identify any volatile compound present. Figure 1 shows the chromatographic profile obtained by the GC/MS technique with the indication of the main identified compounds.

The chemical composition (expressed as % area) of SFE90 determined by GC-MS analysis is reported in Table 1.

The analysis did not reveal the presence of terpenic compounds typically found in essential oils (monoterpene compounds such as α-pinene and β-pinene, limonene, 1,8-cineole or sesquiterpenes such as β-caryophyllene, germacrene D, etc.) [11,15]. However, the following compounds were identified at these concentrations: benzaldehyde, 8.2%; palmitic acid, 9.2%; linoleic acid, 9.6%; and oleic acid, 59.5%.

### 2.3. Analysis of Fatty Acid Profile of Seed Oil Extracts (SFE90, SFE200 and Sx) as Determined by HPLC-DAD/ELSD

**Figure 2:**
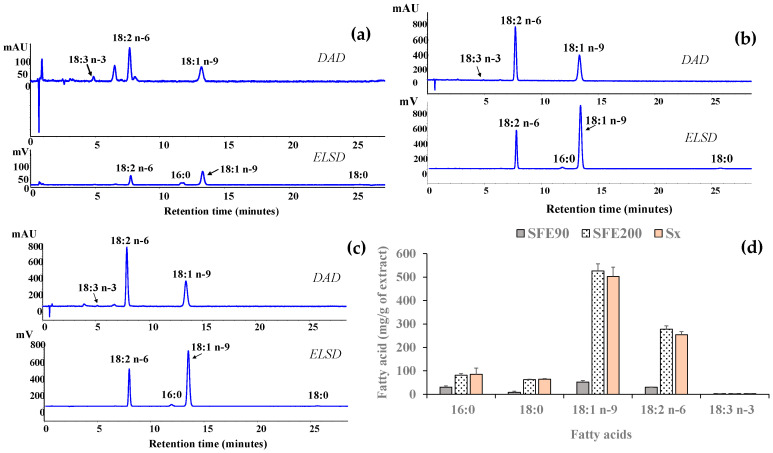
Fatty acid (FA) chromatographic profiles (HPLC analysis) of unsaturated FA (UFA) obtained by DAD detection (200 nm) and saturated FA (SFA) by ELSD detection of *P. spinosa* seed oil extracts SFE90 (**a**) and SFE200 (**b**) obtained by SFE-CO_2_, and Soxhlet extraction (Sx) (**c**). FA composition (expressed as mg/g of oil extract) determined by HPLC-DAD/ELSD analysis of *P. spinosa* SFE90, SFE200 and Sx oil extracts after saponification (**d**). Data are expressed as mean values ± standard deviations (SD) (n = 4).

The chromatographic FA profiles, determined by HPLC-DAD/ELSD analysis, of oil extracts obtained from *P. spinosa* seeds by SFE-CO_2_, SFE90 and SFE200 and the conventional lipophilic solvent *n*-hexane in a Soxhlet apparatus (Sx) are reported in Figures 2a, 2b and 2c, respectively.

Figure 2d shows the FA composition (expressed as mg/g of oil extract) determined by HPLC-DAD/ELSD (Agilent Technologies, Palo Alto, CA, USA) analysis of *P. spinosa* SFE90, SFE200 and Sx oil extracts after mild saponification. The use of two detectors allowed the identification and quantification of saturated FA (SFA, ELSD detection) and unsaturated FA (UFA, UV detection at a wavelength of 200 nm) [10]. SFE200 showed a concentration of 15.2% SFA, mainly palmitic acid 16:0 (8.5%, 81.3 ± 6.8 mg/g of extract) and stearic acid 18:0 (6.6%, 62.8 ± 1.4 mg/g of extract), 55.3% of oleic acid 18:1 n-9 (526.3 ± 29.8 mg/g of extract), and 29.5% of polyunsaturated FA (PUFA), mainly constituted by the essential FA linoleic acid (18:2 n-6) and α-linolenic acid (18:3 n-3), which represented 29.2% (278.0 ± 13.5 mg/g of extract) and 0.3% (2.9 ± 0.2 mg/g of extract) of the FA content, respectively.

The FA composition of SFE200 was similar to that of Sx oil extract obtained with hexane extraction (Figure 2c). The most abundant fatty acids of Sx extract were 18:1 n-9 (502.9 ± 39.2 mg/g of extract, 55.3%), 18:2 n-6 (254.2 ± 13.7 mg/g of extract, 27.9%) and 16:0 (86.0 ± 26.1 mg/g of extract, 9.5%), and the essential fatty acid 18:3 n-3 averaged 2.8 mg/g ± 0.5 mg/g of extract. No significant difference was determined between SFE and Sx.

As observed by GC-MS analysis, oleic acid represented the most abundant UFA after saponification in SFE90 (52.4 ± 6.1 mg/g of extract, 42.1%), followed by 16:0 (30.8 ± 5.2 mg/g of extract, 24.7%) and 18:2 n-6 (29.9 ± 1.3 mg/g of extract, 24.0%), whereas 18:0 (8.9 mg/g ± 4.3 mg/g of extract) and 18:3 n-3 (2.5 mg/g ± 0.6 mg/g of extract) were present in very low amounts. SFE90 showed a significantly lower amount of total FA (124.5 ± 1.7 mg/g of extract) than SFE200 (951.5 ± 42.8 mg/g of extract) and Sx (909.6 ± 46.0 mg/g of extract).

### 2.4. Analysis of Polyphenols in WE90 and WE200

The analysis of phenolic compounds in the two aqueous extracts obtained from the supercritical fluid extraction of *P. spinosa* seeds, WE90 and WE200 revealed both similarities and differences between the two extraction conditions at 90 bar and 200 bar (Table 2).

The phytochemical analysis of *P. spinosa* seed supercritical fluid water extracts (WE90 and WE200) revealed a diverse profile of phenolic compounds including cinnamic acid derivatives, flavanols, flavanones, dihydrochalcones, flavonols, flavones and anthocyanins. The quantification of these compounds was based on the mean intensity abundance relative to the total ion current chromatogram (TIC), enabling a comparative evaluation between the two extraction methods. Notably, certain phenolic compounds demonstrated significant differences in abundance between WE90 and WE200, suggesting that extraction conditions play a critical role in determining phenolic composition.

Among the cinnamic acid derivatives, 3-feruloylquinic acid exhibited the highest intensity (24,929.34 × 10^10^) in WE200, indicating that this compound is preferentially extracted under higher-pressure conditions. Similarly, caffeic acid hexosides displayed high intensities, particularly caffeic acid hexoside 2 with an intensity of 20,220.85 × 10^10^ in WE200. This trend suggests that a higher extraction pressure favors the recovery of certain phenolic acids. In contrast, compounds such as 3-*p*-coumaroylquinic acid showed relatively low intensities in WE90 but increased significantly in WE200 (308.26 × 10^10^), further supporting the influence of extraction conditions on compound recovery.

Flavanols were also detected, with catechin and several procyanidin dimers identified. Catechin was present at higher intensities in WE200 (157.15 × 10^10^) compared to WE90 (31.34 × 10^10^), indicating better extraction efficiency under higher pressure. The presence of multiple procyanidin dimers, although with generally low intensities, suggests that *P. spinosa* seeds contain oligomeric flavanols, which may contribute to the overall antioxidant capacity of the extracts.

The flavonol profile was particularly rich, with numerous quercetin derivatives identified. Notably, quercetin 3′-*O*-glucoside was the most abundant flavonol in WE200 (170.91 × 10^10^) compared to WE90 (34.47 × 10^10^), suggesting that the extraction conditions employed in WE200 are more efficient at recovering glycosylated flavonoids. Furthermore, quercetin-3-*O*-xyloside exhibited high intensities in both extracts, with 9393.80 × 10^10^ in WE200, further supporting the idea that glycosylation enhances the solubility of flavonols under supercritical fluid extraction conditions. Other quercetin derivatives, such as quercetin-3-*O*-glucoside and quercetin-3-*O*-galactoside, were also detected, although at lower intensities.

Flavones such as apigenin pentoside were also identified, with WE90 showing a higher intensity (36.45 × 10^10^) than WE200, indicating that the lower-pressure condition may be more suitable for the extraction of certain flavones. However, most of the identified flavones showed relatively low intensities overall.

Anthocyanins were another major class of compounds identified, with cyanidin derivatives being the most prominent. Cyanidin 3,5-diglucoside (14,275.09 × 10^10^), cya-nidin-3-*O*-rutinoside (16,198.68 × 10^10^) and cyanidin rhamnosyl hexoside (14,159.03 × 10^10^) were among the most abundant anthocyanins detected, particularly in WE200. These results suggest that higher-pressure conditions favor the extraction of anthocyanins, which is consistent with their hydrophilic nature and potentially improved solubility under such conditions. Additionally, several cyanidin conjugates, such as cyanidin-3-*O*-xylosyl rutinoside and cyanidin-3-*O*-galactoside, were detected, further indicating the diversity of anthocyanins present in *P. spinosa* seeds.

Overall, the results demonstrate that the extraction conditions employed in WE200 are generally more effective at recovering phenolic compounds, particularly glycosylated derivatives and anthocyanins. The use of higher pressure appears to enhance the solubility and recovery of these compounds, likely due to improved penetration of the solvent into the plant matrix and increased efficiency in solubilizing phenolic compounds with higher polarity. The presence of numerous glycosylated phenolic compounds suggests that *P. spinosa* seeds possess significant hydrophilic phytochemicals, which are effectively extracted under supercritical fluid water extraction conditions. Moreover, the detection of various quercetin and cyanidin derivatives highlights the potential antioxidant activity of these extracts.

### 2.5. Analysis of Benzaldehyde in WE90 and WE200

The HPLC-DAD (Agilent Technologies, Palo Alto, CA, USA) analysis at 280 nm of the two aqueous extracts (WE90 and WE200) obtained from SFE-CO_2_ extraction of *P. spinosa* seeds revealed the presence of benzaldehyde in these extracts, accounting for 155.1 ± 2.0 μg/mL and 20.5 ± 1.4 μg/mL in WE90 and WE200, respectively.

### 2.6. Cytotoxic Effect of Extracts in Cancer SH-SY5Y Cells and HaCaT Keratinocytes

The cytotoxicity of SFE and water extracts (WE90 and WE200) was investigated, by the MTT viability colorimetric assay, in human neuroblastoma SH-SY5Y cells [16], a cancer cell line, and in human HaCaT keratinocytes [15], spontaneously immortalized cells derived from normal human skin cells.

**Figure 3:**
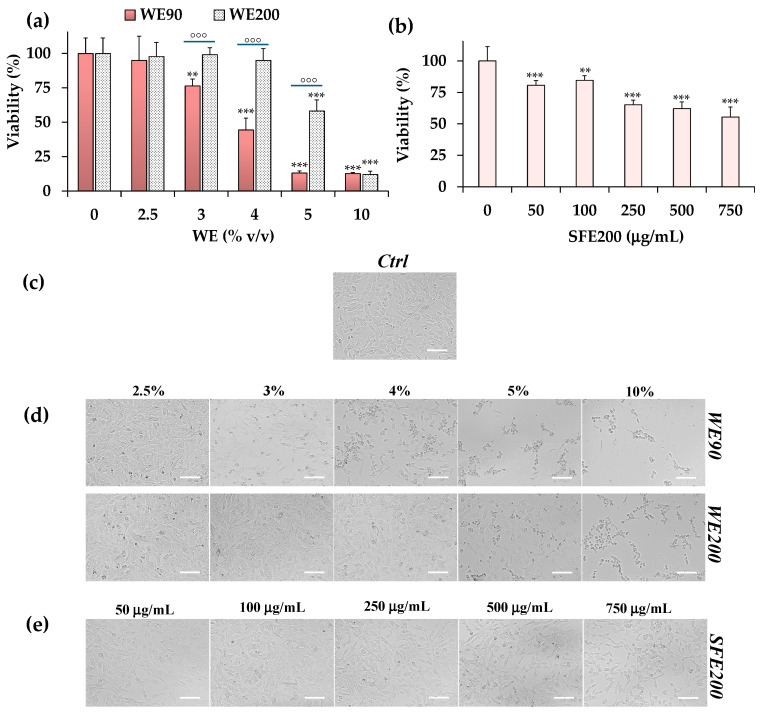
Viability (MTT assay) expressed as % of the control (0), induced by incubation for 24 h with different amounts of *P. spinosa* WE90 and WE200 water extracts (2.5–10% *v*/*v*) (**a**) and SFE oil extract (50–750 μg/mL) (**b**) in human neuroblastoma SH-SY5Y cells. Data are presented as mean and standard deviation (n = 9). For each series, *** = *p* < 0.001, ** = *p* < 0.01 versus respective control (one-way ANOVA and Bonferroni post test). For each concentration group, °°° = *p* < 0.001 for WE90 versus WE200 (Student’s unpaired *t*-test with Welch’s correction). Representative images of phase contrast of control cancer SH-SY5Y cells (**c**) and cells treated with different concentrations of water extracts (WE90 and WE200) (**d**) and SFE oil extract (**e**). Bar = 100 μm.

Figure 3 shows the viability, expressed as % of the control (0), induced by incubation for 24 h with various concentrations of WE90 and WE200 water extracts (2.5–10% *v*/*v*) (Figure 3a) and SFE oil extract (50–750 μg/mL) (Figure 3b) in human cancer SH-SY5Y cells.

WE90 exerted a dose-dependent SH-SY5Y cancer cell growth inhibition from the dose of 3% *v*/*v*, with a significant cell viability reduction versus control (untreated) cells from 24 to 88% in the concentration range of 3–10% *v*/*v* (Figure 3a).

At low doses, WE90 was significantly more toxic in cancer SH-SY5Y cells than WE200. SH-SY5Y cells treated with WE200 showed a significant growth inhibition from the concentration of 5% *v*/*v* (Figure 3a), exhibiting a cell viability reduction versus control cells of 42 and 88% at the doses of 5 and 10% *v*/*v*, respectively.

Microscopic observation of SH-SY5Y cells treated for 24 h with WE90 and WE200 (Figure 3c,d), before the MTT assay, showed evident changes in cell morphologies with respect to control cells. Control neuroblastoma SH-SY5Y cells (untreated) were small and closely packed, while the treatment with *P. spinosa* water extracts induced a reduction in the cell number and a remarkable increase in the number of rounded (apoptotic) cells from the doses of 3% *v*/*v* and 5% *v*/*v* for WE90 and WE200, respectively.

Cells treated for 24 h with the SFE oil extract (Figure 3b) showed a significant viability reduction versus control cells from 19 to 45% at the dose range of 50–750 μg/mL. Microscopic observation of SH-SY5Y cells treated with SFE (Figure 3e) evidenced a reduction in the cell number and changes in cell morphology, with an increase in rounded cells.

DMSO used to dissolve SFE was not toxic in differentiated SH-SY5Y cells after 24 h of incubation, showing a 93% cell viability at the maximal tested dose (1.5%).

**Figure 4:**
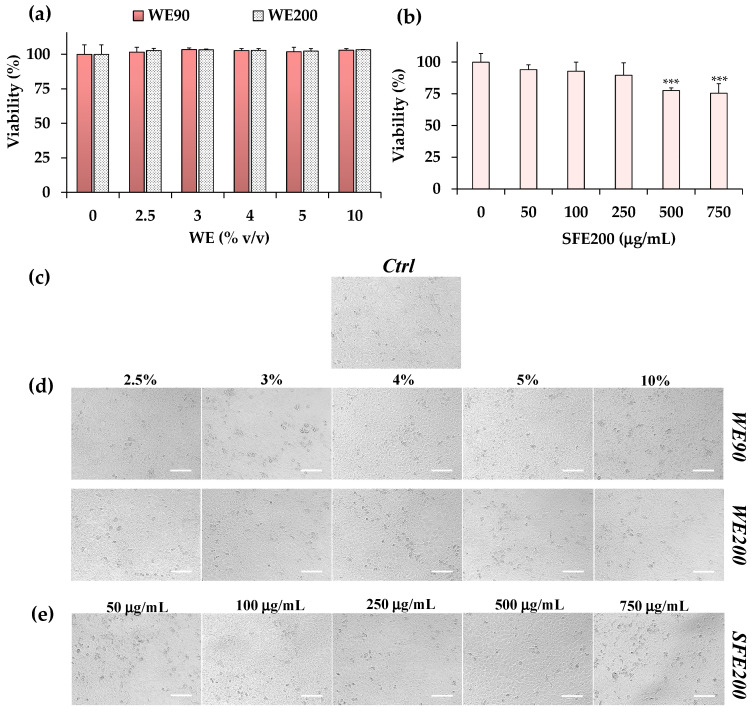
Viability (MTT assay) expressed as % of the control (0), induced by incubation for 24 h with different amounts of *P. spinosa* WE90 and WE200 water extracts (2.5–10% *v*/*v*) (**a**) and SFE oil extract (50–750 μg/mL) (**b**) in human keratinocyte HaCaT cells. Data are presented as mean and standard deviation (n = 9). For each series, *** = *p* < 0.001 versus respective control (one-way ANOVA and Bonferroni post test). Representative images of phase contrast of control HaCaT cells (**c**) and cells treated with different concentrations of water extracts (WE90 and WE200) (**d**) and SFE oil extract (**e**). Bar = 100 μm.

Figure 4 shows the viability, expressed as % of the control, induced by incubation for 24 h with various concentrations of water extracts WE90 and WE200 (2.5–10% *v*/*v*) and SFE oil extract (50–750 μg/mL) in human HaCaT keratinocytes.

WE90 and WE200 did not affect HaCaT cell viability (Figure 4a) and morphology (Figure 4d) in the tested concentration range. A cell growth inhibition was observed in HaCaT keratinocytes treated with SFE200 (Figure 4b) from the dose of 500 μg/mL, with a viability reduction versus control (untreated) cells of 22 and 24% at 500 and 750 μg/mL, respectively.

Microscopic observation of SFE-treated HaCaT cells did not show effects on cell number and morphology (Figure 4e).

## 3. Discussion

There are significant natural sources of bioactive compounds, which can be categorized into plants, algae and food- or agriculture-related by-products. In recent years, interest in plants as potential natural sources has remained strong, with new species continuously being explored. One key area of focus is the utilization of agrifood by-products as sources of valuable compounds. With growing environmental awareness, considerable efforts are being directed toward developments in the circular economy and bioeconomy [20].

Seeds represent the unused part of *P. spinosa* fruits and are commonly considered agro-industrial waste; however, they are considered a rich source of many bioactive components with a wide range of health-promoting properties [1,5,8]. The extraction and recovery of valuable products from *P. spinosa* waste offer a sustainable way to convert waste into wealth.

Conventional extraction methods are simple and cost-effective and have limitations in solvent use, selectivity and product quality, whereas supercritical fluid extraction has emerged as a promising alternative [21].

In this work, two lipid extracts (SFE90 and SFE200) and two aqueous extracts (WE90 and WE200) were obtained from dried seeds of *P. spinosa* by supercritical CO_2_ extraction using different extraction conditions. All extracts were analyzed for chemical composition and biological activity.

The characterization of *P. spinosa* seed extracts obtained through supercritical CO_2_ extraction provides valuable insights into how extraction conditions influence chemical composition and biological activity.

The comparison between low-pressure (90 bar) and high-pressure (200 bar) extraction highlights significant differences in yield and compound selectivity. The supercritical CO_2_ extraction at 200 bar resulted in a higher extraction efficiency and a more complex lipid profile compared to the 90-bar process, reflecting the well-established principle that supercritical CO_2_’s density increases with pressure and, therefore, improves the solubility of oil, and hence results in a higher recovery of seed oil [13,22,23].

GC-MS analyses of volatile constituents of SFE90 evidenced the presence of oleic acid (18:1 n-9) as the main FA, followed by linoleic acid (18:2 n-6) and palmitic acid (16:0). The SFE90 FA profile was confirmed by HPLC-DAD/ELSD analysis after mild saponification; however, SFE90 exhibited a total FA amount lower than SFE200 and Sx.

SFE200, analyzed by HPLC-DAD/ELSD after saponification, showed a composition characterized by a high ratio (approximately 85% of total FA) of UFA to SFA. Oleic acid, the most abundant MUFA, represented the main FA in SFE200, followed by linoleic acid, an essential FA indispensable for human health [24], and palmitic acid.

*P. spinosa* oil extracted using the SFE technique was compared with the oil extracted by *n*-hexane in a Soxhlet apparatus. Soxhlet extraction yielded a significantly higher extraction efficiency; indeed, its continuous solvent reflux may lead to the co-extraction of undesirable compounds, whereas supercritical extraction allows for a more selective recovery of bioactive molecules, minimizing the co-extraction of unwanted substances and leading to a purer final product [25]. Interestingly, the FA composition of SFE200 was similar to that of Sx oil extract obtained with hexane extraction, as previously observed [9].

The FA profile of *P. spinosa* seed oil was comparable to that previously reported in the literature. *P. spinosa* kernel oil, obtained by the cold-press extraction method, has been reported to contain a high content of UFA (91.6%), and oleic acid (72.7%) and linoleic (17.7%) acids have been identified as the main FAs [8]. A high content of oleic acid (43.9%) and a very small amount of SFA like palmitic acid (4.9%) have been quantified in the *P. spinosa* kernel oil obtained by petroleum ether extraction in a Twisselmann apparatus for 6 h [7]. Meanwhile, a higher proportion of PUFA (mainly linoleic acid, 64.4%) compared to MUFA (oleic acid, 29.3%) was reported in *P. spinosa* kernel oil obtained by extraction with *n*-hexane for 6 h [1].

The FA composition is important in terms of nutritional/functional characteristics of the oils. The FA profile of SFE200, dominated by oleic acid 18:1 n-9, suggests a strong potential nutritional application of this fixed oil, as this MUFA is widely recognized for its beneficial health properties [26]. MUFA-rich food has been suggested to beneficially modulate the blood lipid profile [27]. The high oleic and linoleic acid contents of SFE200, in conjunction with its low level of SFA, mean this oil is of exceptional interest in terms of promoting human health [1,7,24,26,27].

Regarding SFE90, WE90 and WE200 composition, it is interesting to underline the presence of benzaldehyde. Literature data report that cyanogenic glucosides are commonly found in various Prunus species as well as in numerous other Rosaceae [1,5,28,29]. Cyanogenic glucoside undergoes enzymatic hydrolysis into its constituents—including benzaldehyde—when in contact with specific enzymes stored in separate cellular compartments from the compound itself [5,30,31]. It can be inferred that the presence of benzaldehyde in SFE90, WE90 and WE200 obtained from *P. spinosa* was probably due to enzymatic hydrolysis of cyanogenic glycosides (amygdalin and prunasin) present in the seeds, as previously demonstrated in the literature [1,5,30,31].

The analysis of polyphenolic compounds in WE90 and WE200 highlighted the impact of pressure on compound solubility and extraction efficiency. The enrichment of caffeic acid hexoside 2 and dihydro-*o*-coumaric acid glucoside in WE200 suggests that increased pressure enhanced the extraction of specific hydroxycinnamic acid derivatives, whereas lower pressure allowed for a broader range of compounds. These findings align with previous reports on *P. spinosa*, which indicate that hydroxycinnamic acids and their derivatives play a major role in the antioxidant capacity of the extracts [3,32]. The distribution of flavanols, flavanones and flavonols further supports this observation, with high-pressure extraction favoring flavonol glycosides, particularly quercetin derivatives, while low-pressure extraction retained more flavanones and dihydrochalcones [33].

The anthocyanin composition varied significantly between extraction conditions, with WE200 favoring cyanidin-3-sambubioside and peonidin-3-*O*-glucoside, while WE90 was richer in cyanidin-3-*O*-rutinoside and cyanidin-3,5-diglucoside. This shift indicated that lower pressure enhanced the extraction of diglucosides and rutinosides, compounds often associated with greater stability and bioaccessibility [26]. These results are consistent with previous studies highlighting the role of anthocyanins in *P. spinosa* fruits and their correlation with antioxidant and antiproliferative activity [32,33].

Several recent studies have shown that polar extracts isolated from *P. spinosa* L. fruits have beneficial cytotoxic activity on some cancer cell lines, due to their high content of phenolic acids and flavonoids [33,34,35]. Moreover, we previously demonstrated the ability of oil extracts obtained by SFE-CO_2_ at high pressure from *Lycium europaeum* fruits and *Aronia melanocarpa* berries to induce cytotoxicity in cancer cells [10,15]. Therefore, WE90, WE200 and SFE200 were evaluated for their ability to affect viability in human cancer SH-SY5Y cells, a cultured cancer cell model extensively used in the prediction of the potential antitumor properties of natural compounds/extracts and to study neuroblastoma-specific therapeutics [16,36], and in human keratinocyte HaCaT cells, amply used as a skin cell model to test natural extracts [15,37].

Both aqueous extracts affected viability and morphology in cancer cells. However, the cytotoxicity analysis of WE90 and WE200 in SH-SY5Y neuroblastoma cells revealed notable differences. At low doses, WE90 was significantly more toxic in cancer SH-SY5Y cells than WE200, inducing a more marked reduction in the cell number and increase in the number of rounded (apoptotic) cells.

This suggests that the polyphenolic composition, rather than the total polyphenol content, plays a critical role in determining WE bioactivity. The presence of specific anthocyanins and flavonoids in WE90 probably contributed to its stronger inhibitory effect on cancer cell growth, while WE200 required a higher concentration to achieve similar results. These findings align with previous research on *P. spinosa*, which correlated anthocyanin and flavonoids content with antiproliferative effects, particularly in colon carcinoma cells [3,35,38].

Moreover, the higher amount of benzaldehyde in WE90 than WE200 could contribute to the significant difference in cytotoxicity. Previous studies evidenced the capacity of benzaldehyde and its inclusion compound to induce cell mortality in cancer cells [39,40].

The 24 h treatment with oil extract SFE200 induced a significant viability reduction versus control cells, coupled with a reduction in the cell number and marked changes in cell morphology.

Interestingly, WE extracts did not affect HaCaT cell viability and morphology in the tested concentration range, while a slight cell growth inhibition was observed only in keratinocytes treated with the highest doses of SFE200. The selective cytotoxicity observed in cancer cells, without affecting human skin keratinocytes, further supports the potential application of *P. spinosa* extracts in functional and nutraceutical formulations targeting cancer prevention and treatment.

The findings of this study reinforce the importance of extraction parameters in deter-mining the composition and biological properties of *P. spinosa* seed extracts. For WE, higher pressure enhanced the recovery of specific flavonols and anthocyanins, while lower pressure preserved a more diverse range of polyphenolic subclasses with distinct bioactivities. Meanwhile, for lipophilic compounds, high pressure allowed us to obtain a fixed oil rich in oleic and linoleic acids. These results contribute to the growing body of evidence supporting the bioactive potential of *P. spinosa* and its possible nutritional, nutraceutical and pharmaceutical applications, particularly for its antioxidant and anticancer properties. Further studies focusing on bioavailability and in vivo efficacy will be essential to fully exploit the therapeutic potential of these extracts.

## 4. Materials and Methods

### 4.1. Chemicals and Reagents

Standards of fatty acids, 3-(4,5-dimethylthiazol-2-yl)-2,5-diphenyltetrazolium bromide (MTT), benzaldehyde and all solvents used (purity ≥ 99.9%) were obtained from Sigma-Aldrich (Milan, Italy). Cell culture materials were acquired from EuroClone (Pero, MI, Italy). All the chemicals used in this study were of analytical grade.

### 4.2. Plant Material

The whole and dried fruits were purchased from the Minardi company (Bagnacavallo, RA, Italy), Lot N° G-120923-12, from Serbia, collected from the wild in 2023. From receipt, the matrix was stored in a cool and dry place at a temperature not exceeding 25 °C. To prepare the feed to be extracted, the fruits were cut in two and the seeds separated from the pulp. The seeds were cleaned and their surface hair removed. Before utilization, the vegetable matter was ground and reduced to powder with a Malavasi mill (Bologna, Italy), taking care to avoid overheating. The material destined for SFE was split into two lots, using mechanical sieving, with particle sizes in the ranges of 250–425 and >850 μm, respectively.

### 4.3. Supercritical Carbon Dioxide Extraction

Supercritical carbon dioxide extraction (CO_2_ purity, *v*/*v* > 99.7% in 30 kg cylinders equipped with a dip tube, supplied by Air Liquide-Italy) was performed in a laboratory apparatus equipped with a 320 cm^3^ extraction vessel. Extractions were carried out in a semi-batch mode, as previously described [9,11,15].

The extracts in question were obtained, starting only from seeds finely ground, using the plant just described with the single separator setup. The operating conditions were as follows: pressure of 90 or 200 bar and temperature of 40 °C, in the extraction section, and 20 bar and 40 °C in the separation section; extraction time of 4 h; and CO_2_ flow rate of 1.2 kg h*^−^*^1^. At 200 bar and 40 °C, the CO_2_ has a high density and solvent power, while at 20 bar and 40° C, the CO_2_ returns to the state of sub-critical gas, loses its solubilizing power and releases the extract to the inside the separator. By opening the lower valve of the separator, it is possible to collect the final extract, from which the gaseous CO_2_ at ambient pressure and temperature moves away spontaneously. In each of the tests carried out, an average of 300 g of matrix was loaded into the extractor. The extraction at 200 bar was run on the same sample previously treated at 90 bar.

### 4.4. Soxhlet Extraction

Solvent extraction was performed with n-hexane in a Soxhlet apparatus to compare the extraction performances with SFE-CO_2_. The extraction lasted 6 h at the boiling temperature of the solvent. n-Hexane evaporation was performed under vacuum on a rotary evaporator at 40 °C to obtain the extract Sx. The samples intended for chemical and biological characterization were stored at +4 °C until their use.

### 4.5. SFE90 Analysis by GC-MS

SFE 90 analysis was carried out by gas chromatography/mass spectrometry (GC-MS), using a gas chromatograph (Agilent 6890N, Santa Clara, CA, USA) equipped with a 30 m × 0.25 mm i.d. with 0.25 µm stationary film thickness HP-5ms capillary column (Agilent J&W) coupled with a mass selective detector with an electron ionization device, EI and quadrupole analyzer (Agilent 5973). The following temperature program was used: from 60 °C to 246 °C at a rate of 3 °C min^−1^ and then held at 246 °C for 20 min (total analysis time 82 min). Other operating conditions were the following: carrier gas, helium (purity ≥ 99.9999%, Air Liquid, Assemini, Italy); flow rate, 1.0 mL/min; injector temperature, 250 °C; detector temperature, 300 °C. Injection of 1 μL of diluted sample (1:100 in n-hexane, *w*/*w*) was performed with a 1:20 split ratio, using an autosampler (Agilent, Model 7683B).

The MS conditions were as follows: MS transfer line temperature, 240 °C; EI ion source temperature, 200 °C with ionization energy of 70 eV; quadrupole temperature, 150 °C; scan rate, 3.2 scan s-1 at *m*/*z* scan range (30 to 480). The software MSD ChemStation G1701EA (rev. E.01.00.237, Agilent Technologies) was used to handle and process chromatograms and mass spectra. Compounds were identified by comparison of their mass spectra with those of NIST02 library data [19] of the GC/MS system and Adams libraries spectra [18]. The results were further confirmed by comparison of the compound elution order with their retention indices on semi-polar phases reported in the literature [18]. Retention indices of the components were determined relative to the retention times of a series of n-alkanes (two standard mixes C8–C20 and C21–C40) with linear interpolation [41]. The percentage of individual components was calculated based on GC peak areas without FID response factor correction.

### 4.6. Determination of Fatty Acid Profiles of Oil Extracts (SFE90, SFE200 and Sx) by HPLC-DAD/ELSD Analysis

Aliquots (1.5 mg in ethanol solution) of SFE200 and Sx oil extracts obtained from *P. spinosa* seeds were subjected to mild saponification, as previously reported [10]. The mixtures were left in the dark at room temperature for 14 h. Then, after the addition of n-hexane and H_2_O, samples were acidified to pH 3–4 with 37% HCl and centrifuged (1 h at 900× *g*). The hexane phase (saponifiable fraction) containing free FA was collected and the solvent was evaporated under vacuum. The dried residues, dissolved in acetonitrile, were then injected into an Agilent Technologies 1100 HPLC system equipped with a DAD and an Agilent Technologies Infinity 1260 evaporative light scattering detector (ELSD), for the FA analysis, as reported [10,15]. Analyses of unsaturated (DAD detection, 200 nm) and saturated (ELSD detection) FA, obtained from saponification, were carried out with a mobile phase of acetonitrile/water/acetic acid (75/25/0.12, *v*/*v*/*v*), at a flow rate of 2.3 mL/min. Collected data were analyzed using the Agilent OpenLAB Chromatography data system, as previously described [10,15]. Calibration curves of FA were constructed using standards and were found to be linear (DAD) and quadratic (ELSD) (correlation coefficients > 0.995) [10,15].

### 4.7. LC-DAD-ESI-MS Analysis of WE90 and WE200

The phytochemical composition of *P. spinosa* water extracts was investigated through LC-DAD-ESI-MS analysis. Separation was performed using a Luna Omega PS C18 column (150 mm × 2.1 mm, 5 μm; Phenomenex, Torrance, CA, USA) maintained at 25 °C. The mobile phase consisted of 0.1% formic acid and acetonitrile, following the elution protocol described by Danna et al. [42]. Each extract (5 μL) was injected, and the UV–Vis spectra of the analytes were recorded across a wavelength range of 190–600 nm. Chromatographic data were collected at 260, 292, 330, 370 and 520 nm to ensure the detection of various polyphenol classes. Mass spectrometric analysis was conducted using an ion trap (model 6320, Agilent Technologies, Santa Clara, CA, USA) operating in full-scan mode (90–1000 *m*/*z*) under both positive and negative electrospray ionization (ESI) conditions, as outlined by Danna et al. [41]. Compound identification was achieved by comparing retention times, UV–Vis spectra and mass spectra of the detected analytes with commercially available HPLC-grade standards (see Table 2 footnote for details), as well as with literature data and free online consulting UV–Vis and mass spectra databases (SpectraBase^®^, PhytoHub, ReSpect for Phytochemicals, Mass Bank and PubChem). The results, from three independent triplicate analyses (n = 3), are expressed as the mean intensity abundance of each compound relative to the total ion current chromatogram (TIC). For compounds detected in both positive and negative ionization modes, the reported intensity values were selected based on three criteria: signal intensity (signal-to-noise ratio) and data consistency (repeatability and reliability across replicates). When significant differences in signal intensity were observed between the two modes, the mode providing the higher and more reliable signal was preferentially reported.

### 4.8. Quantification of Benzaldehyde in WE90 and WE200 by HPLC-DAD Analysis

Aliquots (1 μL) of WE90 and WE200 extracts obtained from *P. spinosa* seeds were directly injected into an Agilent Technologies 1100 HPLC system equipped with a DAD detector for the analysis of benzaldehyde. The analysis was carried out with a mobile phase of acetonitrile/water (10/90, *v*/*v*), acidified with orthophosphoric acid (1% *v*/*v*), at a flow rate of 1 mL/min (at 280 nm), and data were analyzed using the Agilent OpenLAB Chromatography data system. A benzaldehyde standard was used for the construction of the calibration curve (linear correlation coefficient = 0.9999).

### 4.9. Cell Cultures

The human HaCaT keratinocyte cell line, spontaneously immortalized cells derived from normal human skin cells, was obtained from CLS-Cell Line Services (Eppelheim, Germany). Human neuroblastoma cell line SH-SY5Y (code# HTL95013) was supplied by the Cell Bank Interlab Cell Line Collection, IRCCS San Martino Policlinico Hospital (Genova, Italy). Cell lines were grown at 37 °C in Dulbecco’s modified Eagle’s medium (DMEM) with high glucose, enriched with 2 mM L-glutamine, penicillin (100 units/mL)–streptomycin (100 μg/mL) and 10% *v*/*v* fetal calf serum (FCS), in an atmosphere of 5% CO_2_ and 95% humidity. The cells were split once a week before they reached confluency using a trypsin 0.25%-EDTA solution.

### 4.10. Cytotoxic Activity: MTT Assay

The MTT colorimetric assay was used to assess the cytotoxic effect of SFE oil extract and water extracts WE90 and WE200 in SH-SY5Y neuroblastoma cells and HaCaT cells, as previously reported [15,16]. SH-SY5Y neuroblastoma and HaCaT cells were plated in a 96-well plate at 10^5^ cells/mL density in 100 μL of complete culture medium and cultured for 48 h. After medium removal, cells (at 80–90% cell confluence) were incubated for 24 h with various concentrations of water extracts WE90 and WE200 (2.5–10% *v*/*v*) and SFE200 oil extract (50–750 μg/mL, from a solution 40 mg/mL in DMSO) in fresh medium (treated cells). Treated cells were compared for viability to control cells (non-treated). After incubation, cells were subjected to the MTT viability test, as previously reported [15,16]. Color development (absorbance proportional to the number of viable cells) was measured at 570 nm with an Infinite 200 auto microplate reader (Infinite 200, Tecan, Austria). Results were expressed as the percentage of cell viability with respect to control cell viability (100%). Evaluation of the cell morphology after 24 h of incubation with various amounts of extracts was performed by microscopic analysis with a ZOE™ Fluorescent Cell Imager (Bio-Rad Laboratories, Inc., Hercules, CA, USA).

### 4.11. Statistical Analyses

Evaluation of the statistical significance of differences was performed using Graph Pad INSTAT software v3.0 (GraphPad Software, San Diego, CA, USA). Results were expressed as mean ± standard deviation (SD), and statistically significant differences were evaluated with *p* < 0.05 as a minimal level of significance. Multiple comparisons of means were assessed by one-way analysis of variance (one-way ANOVA) followed by the Bonferroni multiple comparisons test to substantiate statistical differences between groups. Student’s unpaired *t*-test with Welch’s correction was used to compare the means of two groups.

## 5. Conclusions

This study highlights the impact of supercritical extraction pressure on the yield, composition and bioactivity of *P. spinosa* seed extracts. Higher pressure enhanced extraction efficiency and favored flavonol glycosides, while lower pressure retained a broader range of flavonoids, including flavanones and flavones. A shift in anthocyanin composition was observed, with WE90 enriched in cyanidin-3-sambubioside and WE200 dominated by cyanidin-3-rutinoside. Cytotoxicity assays revealed that WE90 exhibited stronger anticancer effects in SH-SY5Y neuroblastoma cells, while neither extract strongly affected viability in human skin HaCaT keratinocytes. The fatty acid profile was similar between supercritical and Soxhlet-extracted oils, with oleic and linoleic acids as the main components.

These findings confirm the potential of *P. spinosa* seed extracts for nutraceutical and pharmaceutical applications, particularly for antioxidant and anticancer properties.

Further studies on bioavailability and in vivo efficacy are needed to support their functional use.

## Figures and Tables

**Table 1:** Tentative identification of volatile compounds of *P. spinosa* seed extract obtained by supercritical CO_2_ extraction at low pressure (SFE90). Results were measured by GC-MS analysis and expressed as mean area percentage (%).

Number	t_R_ (min)	RI_EXP_^1^	RI_LIT_	Compound	% Area	Formula
1	6.205	961	952	Benzaldehyde	8.2	C_7_H_6_O
2	8.396	1031		n.i.	1.8	
3	44.356	1961	1959	Palmitic acid	9.2	C_16_H_32_O_2_
4	49.588	2129	2132	Linoleic acid	9.6	C_18_H_32_O_2_
5	49.883	2139	2141	Oleic acid	59.5	C_18_H_34_O_2_
6	50.220	2151		n.i.	2.1	
7	50.438	2158		n.i.	6.7	
8	57.788	2420		n.i.	0.4	
9	62.995	2621		n.i.	0.3	
10	68.033	2796		n.i.	2.4	

^1^ RI_EXP_, retention index determined on a HP-5ms fused silica column relative to a series of n-alkanes. Compounds were identified by comparing their retention indices (RI_EXP_) and mass spectra (MS) with those reported in Adams (RI_LIT_) [18] and NIST02 [19] libraries. n.i. = not identified.

**Table 2:** Tentative identification of phenolic compounds in *P. spinosa* seed supercritical fluid water extracts (WE90 and WE200) using LC-DAD-ESI-MS (Agilent Technologies, Santa Clara, CA, USA) analysis. The results are expressed as the mean intensity abundance of each compound relative to the total ion current chromatogram (TIC).

Compounds	MW	[M + H]^+^	[M − H]^−^	WE90	WE200
		**(*m*/*z*)**	**(*m*/*z*)**	**Intensity (×10^10^)**	
*Cinnamic acid derivatives*					
3-Caffeoylquinic acid	354		353	2.25	
Caffeic acid hexoside 1	342		341		81.96
Caffeic acid hexoside 2	342	343			20,220.85
Dicaffeoylquinic acid	516		515		38.87
4-*p*-Coumaroylquinic acid	338		337	2.26	
*o*-Coumaric acid diglucoside	488		487		25.92
4-*O*-Caffeoylquinic acid	354		353	27.10	
1-Caffeoyl-5-feruloylquinic acid	530		529	3.98	
3-*p*-Coumaroylquinic acid	338		337	39.61	308.26
4-*p*-Coumaroylquinic acid 1	338	339		7175.09	
4-*p*-Coumaroylquinic acid 2	338	339		5370.64	
3-*p*-Coumaroyl 5-caffeoylquinic acid	500	501		10,124.89	
3-Feruloylquinic acid	368	369		24,929.34	
Dihydro-*o*-coumaric acid glucoside	328	329			19,167.56
*Flavanols*					
Catechin ^a^	290		289	31.34	157.15
Procyanidin dimer 1	578		577	1.43	
Procyanidin dimer 2	578		577	1.49	18.32
Procyanidin dimer 3	578		577	1.88	
Procyanidin dimer 4	578		577	5.21	
*Flavanones*					
Naringenin hexoside 1	434	435	433		5446.25
*Dihydrochalcones*					
Phloridzin ^a^	436		435	5.47	
*Flavonols*					
Quercetin 4′-*O*-glucoside	464		463	1.44	
Quercetin rhamnosylhexoside	610	611	609	3898.20	35.66
Quercetin acetyl rutinoside	652		651	13.21	79.05
Quercetin ^a^	302		301	6.38	59.03
Quercetin 3′-*O*-glucoside	464		463	34.47	170.91
Quercetin acetyl hexoside	506		505	3.23	
Quercetin rhamnosyl hexoside rhamnoside	756		755	9.10	
Quercetin rhamnoside	448		447	31.58	
Quercetin pentoside rhamnosyl hexoside	742		741	41.78	
Quercetin triglycoside	772	773		832.69	
Quercetin-3-*O*-xyloside	434	433		9393.80	
Quercetin hexosyl pentoside 1	596	597			8904.04
Quercetin hexosyl pentoside 2	596	597			8268.87
Quercetin hexosyl pentoside 3	596	597		7911.02	
Quercetin dihexoside	626	627		8352.75	
Quercetin-3-*O*-galactoside ^a^	464	465			3950.81
Quercetin-3-*O*-glucoside ^b^	464	465			4296.66
Isorhamnetin 7-*O*-rhamnoside	478		477	1.29	
Isorhamnetin 3-*O*-glucoside ^a^	478		477	1.36	
Isorhamnetin-3-rutinoside ^a^	624		623	11.08	
Isorhamnetin hexoside	478		477	45.26	
Kaempferol ^a^	286		285	3.17	
Kaempferol 7-*O*-glucoside ^a^	448		447	1.30	20.33
Kaempferol hexoside pentoside	579		578	1.31	
Kaempferol 3-*O*-rhamnoside	431		430	1.38	
Kaempferol 3-*O*-(6″-malonyl-glucoside)	534		533	22.36	
Kaempferol pentoside	418		417	18.46	
Kaempferol hexosyl dirhamnoside	740		739		165.97
Kaempferol rhamnosyl hexoside 1	594		593	29.89	
*Flavones*					
Apigenin pentoside	402		401		36.45
Apigenin rhamnoside	416		415	3.21	
Apigenin 7-*O*-apiosyl-glucoside	564		563	3.24	
*Anthocyanins*					
Cyanidin 3-acetylglucoside	490	491		640.73	608.68
Cyanidin 3,5-diglucoside	610	611		14,275.09	
Cyanidin-3-*O*-rutinoside ^a^	594	595		16,198.68	
Cyanidin rhamnosyl hexoside	594	595		14,159.03	
Cyanidin-3-*O*-xylosyl rutinoside	726	727		4143.21	
Cyanidin-3-*O*-galactoside ^a^	448	449		3838.37	
Cyanidin pentoside	418	419	417	14,118.31	
Cyanidin-3-*O*-sambubioside	580	581	579	3.39	10,472.48
Cyanidin 3-*O*-(6″-malonyl-glucoside)	549		548	1.30	
Cyanidin 3-*O*-(6″-succinyl-glucoside)	549		548	1.63	
Cyanidin 3-*O*-(6″-acetyl-glucoside)	491		490	3.98	
Peonidin-3-*O*-rutinoside ^a^	608	609	607	1.40	
Peonidin-3-*O*-glucoside ^a^	462	463	461	4.81	5601.91
Peonidin 3-*O*-galactoside ^a^	432		461	4.83	
Peonidin 3-*O*-(6″-acetyl-glucoside)	505		504		
Petunidin-3-*O*-rhamnoside	462	463		3.37	
Pelargonidin-3-*O*-glucoside ^b^	432	433	431	3212.70	
Pelargonidin 3-*O*-sambubioside	565		564	9747.23	
Delphinidin 3-*O*-xyloside	435		434	2.82	

^a,b^ Verified using commercially available HPLC-grade reference standards (purity ≥ 98%) obtained from Extrasynthase (Genay, France) and Merck KGaA (Darmstadt, Germany), respectively.

## Data Availability

The datasets generated and analyzed during the current study are available from the corresponding author on reasonable request.
